# *Escherichia coli *BdcA controls biofilm dispersal in *Pseudomonas aeruginosa *and *Rhizobium meliloti*

**DOI:** 10.1186/1756-0500-4-447

**Published:** 2011-10-26

**Authors:** Qun Ma, Guishan Zhang, Thomas K Wood

**Affiliations:** 1 Department of Chemical Engineering, Texas A & M University, College Station, TX 77843-3122, USA

## Abstract

**Background:**

Previously we showed that BdcA controls *Escherichia coli *biofilm dispersal by binding the ubiquitous bacterial signal cyclic diguanylate (c-di-GMP); upon reducing the concentration of c-di-GMP, the cell shifts to the planktonic state by increasing motility, decreasing aggregation, and decreasing production of biofilm adhesins.

**Findings:**

Here we report that BdcA also increases biofilm dispersal in other Gram-negative bacteria including *Pseudomonas aeruginosa*, *Pseudomonas fluorescens*, and *Rhizobium meliloti*. BdcA binds c-di-GMP in these strains and thereby reduces the effective c-di-GMP concentrations as demonstrated by increases in swimming motility and swarming motility as well as by a reduction in extracellular polysaccharide production. We also develop a method to displace existing biofilms by adding BdcA via conjugation from *E. coli *in mixed-species biofilms.

**Conclusion:**

Since BdcA shows the ability to control biofilm dispersal in diverse bacteria, BdcA has the potential to be used as a tool to disperse biofilms for engineering and medical applications.

## Background

Bacteria prefer to grow as biofilms [1]; i.e., as aggregates of bacteria that form usually at interfaces. Cells in biofilms are protected and held together by the extracellular matrix that includes polysaccharide, lipids, protein, and DNA [2]. Biofilms are notoriously difficult to remove [3,4] and are related to 80% of all human infections [5]. In addition, biofilm formation leads to significant energy losses in heat exchange equipment [6] and causes operational problems in the oil industry via metal corrosion, flow reduction, contamination, and reservoir plugging [7]. Conversely, biofilms are becoming increasingly important for chemical transformations; for example, biofilms lead to better productivity for biofuels such as ethanol [8], and biofilm communities form on electrodes surfaces to produce power for microbial fuel cells [9]. Also, for biorefineries, it will be important to be able to remove biofilms from fixed film reactors so that diverse products may be synthesized [10]. Hence, effective strategies to remove biofilms for medical applications as well as to control biofilm formation for engineering applications are important. Biofilm development is a dynamic process that includes movement to the surface [11], initial reversible attachment, irreversible attachment, formation of small aggregates, maturation of the biofilm, and biofilm dispersal [12]. Biofilm dispersal is controlled by various factors, including external forces and the physiological changes of the bacteria themselves [4].

Previously we discovered the biofilm dispersal protein BdcA in *Escherichia coli*, which works by binding to the intracellular signal cyclic diguanylate (c-di-GMP) but does not act as a phosphodiesterase [13]. Reducing c-di-GMP concentrations increases motility and extracellular DNA production and decreases extracellular polysaccharide production (EPS), cell length, and aggregation which leads to biofilm dispersal [13]. We also performed protein engineering on this protein and successfully abolished biofilm formation via single amino acid replacement at E50Q [13]. Hence biofilm dispersal can be achieved by simply by producing BdcA in *E. coli*. However, it has not been demonstrated that this protein is functional and can cause dispersal of biofilms in other bacteria.

Since BdcA works through c-di-GMP [13], and c-di-GMP exists in almost all bacteria [10] and regulates biofilm formation by controlling cell motility in Gram-negative bacteria [14], we hypothesized that BdcA may also work in most Gram-negative bacteria. Hence, here we constructed a broad-host plasmid to produce BdcA (pMMB206-BdcA) and tested the effect of BdcA production on biofilm dispersal for the opportunistic pathogen *Pseudomonas aeruginosa *(responsible for chronic cystic fibrosis infections [15,16]), the biocontrol agent *Pseudomonas fluorescens *(utilized for protecting plants from fungi infection [17,18]), as well as the nitrogen-fixing bacterium *Rhizobium meliloti *[19,20]. For all three bacteria, we find that BdcA is an effective tool for dispersing biofilms.

## Methods

### Bacterial strains, media, and growth conditions

The strains and plasmids used in this study are listed in Table 1. Expression of *bdcA *was induced by 0.1 mM isopropyl-β-*D*-thiogalactopyranoside (IPTG) (Sigma, St. Louis, MO). Luria-Bertani (LB) [21] was used for all the experiments. Chloramphenicol (50 μg/mL for *R. meliloti *and 250 μg/mL for *Pseudomonas *sp.) was used for strains harboring pMMB206 and pMMB206-BdcA. Carbenicillin (50 μg/mL) was used for strains harboring pP25-gfp. 37°C was used for all *P. aeruginosa *tests, and 30°C was used for *P. fluorescens *and *R. meliloti *tests.

**Table 1:** Strains and plasmids used in this study

Strain/Plasmid	Genotype	Source
**Strain**		
*E. coli *BW25113	*lacI*^q ^*rrnB*_T14 _*ΔlacZ*_WJ16 _*hsdR514 ΔaraBAD*_AH33 _*ΔrhaBAD*_LD78_	[34]
*E. coli *DH5α	*luxS supE44 ΔlacU169(Φ80 lacZΔM15) hsdR17 recA1 endA1 gyrA96 thi-1 relA1*	[21,35]
*E. coli *DH5α*/*pMMB206/pRK2013	*E. coli *harboring pMMB206 and pRK2013	This study
*E. coli *DH5α*/*pMMB206-BdcA/pRK2013	*E. coli *harboring pMMB206-BdcA and pRK2013	This study
*P. aeruginosa *PA14	*P. aeruginosa *wild-type strain	[36]
*P. fluorescens*	*P. fluorescens *wild-type strain	R. Frazee
*R. meliloti *102F34	*R. meliloti *wild-type strain	M. Sadowski
PA14/pMMB206	*P. aeruginosa *wild-type harboring pMMB206	This study
PA14/pMMB206-BdcA	*P. aeruginosa *wild-type harboring pMMB206-BdcA	This study
PA14**/**pMMB206/pP25-gfp	*P. aeruginosa *wild-type harboring pMMB206 and pP25-gfp	This study
PA14**/**pMMB206-BdcA/pP25-gfp	*P. aeruginosa *wild-type harboring pMMB206-BdcA and pP25-gfp	This study
*P. fluorescens*/pMMB206	*P. fluorescens *wild-type harboring pMMB206	This study
*P. fluorescens*/pMMB206-BdcA	*P. fluorescens *wild-type harboring pMMB206-BdcA	This study
*R. meliloti *102F34/pMMB206	*R. meliloti *wild-type harboring pMMB206	This study
*R. meliloti *102F34/pMMB206-BdcA	*R. meliloti *wild-type harboring pMMB206-BdcA	This study
*R. meliloti *102F34/pMMB206/pP25-gfp	*R. meliloti *wild-type harboring pMMB206 and pP25-gfp	This study
*R. meliloti *102F34/pMMB206-BdcA/pP25-gfp	*R. meliloti *wild-type harboring the pMMB206-BdcA and pP25-gfp	This study
**Plasmid**		
pMMB206	Cm^r^, Mob^+^, *lacI^q^*, broad-host range plasmid	[23]
pMMB206-BdcA	Cm^r^; pMMB206 with *P_tac-lacUV5_::bdcA*	this work
pRK2013	Km^r^; Tra^+ ^Mob^+ ^(RK2) Km::Tn7 ColEl origin, helper plasmid for mobilization	M. Bagdasarian, [37]
pP25-gfp	Car^r^; constitutive green fluorescent protein production	[27]

Cm^r^, Car^r^, and Km^r^, denote chloramphenicol, carbenicillin, and kanamycin resistance, respectively.

### Plasmid construction

**Figure 1:**
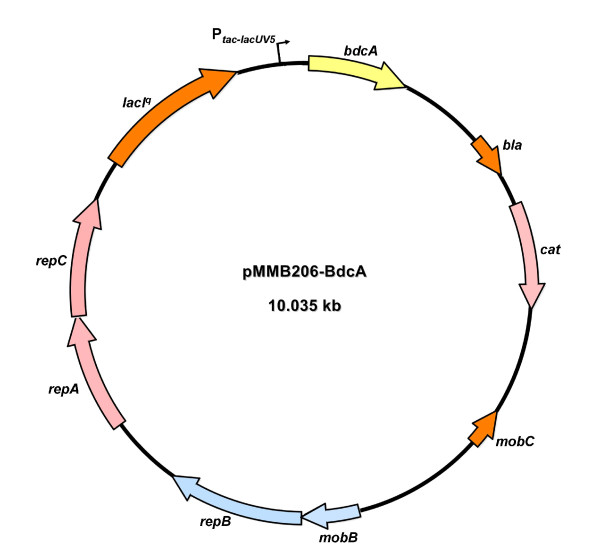
**Plasmid map for pMMB206-BdcA**. The *bdcA *gene is shown in yellow.

The *bdcA *gene was amplified from *E. coli *BW25113 using bdcAc f-primer (5'-CGCGCAGGATCCCACCATCACCACCATCATGGCGCTTTTACAGGTAAGACAGTTC-3') and bdcAc-r primer (5'-GATGTGGAGTCTGCTGAGCTGCAGTTATGCGCCAAACGCGCCATCAATG-3'), and the PCR product was cleaned with the QIAquick PCR purification kit (QIAGEN, Valencia, CA). The *bdcA *PCR product and the pMMB206 vector were both digested with BamHI (New England Biolabs, Beverly, MA) and PstI (New England Biolabs) overnight at 37°C. The vector was treated with alkaline phosphatase (New England Biolabs), and ligation was performed with T4 DNA ligase (New England Biolabs) overnight at 16°C. The ligation product was electroporated into *E. coli *DH5α, and white colonies after blue/white screening were selected and sequenced with sequencing primers bdcAseq f-primer (5'-ACACTTTATGCTTCCGGCTCGTATG-3') and bdcAseq r-primer (5'-GCGATTAAGTTGGGTAACGCCAG-3'). The correctly constructed pMMB206-BdcA (Figure 1) was introduced into the *Rhizobium *strain via electroporation and into the *Pseudomonas *strains via conjugation as previously described [22]. pMMB206-BdcA is a low-copy-number plasmid, and the expression of *bdcA *is under the control of the tandem *tac-lacUV5 *promoter [23].

### Static biofilm assay using crystal violet

Biofilm formation for the individual single strains was assayed in 96-well polystyrene plates using 0.1% crystal violet staining (Corning Costar, Cambridge, MA) as described previously [24] with small modifications. Briefly, each well was inoculated with overnight cultures at an initial turbidity at 600 nm of 0.05 and grown without shaking for 24 h with chloramphenicol, and then 0.1 mM IPTG was added to the culture to induce *bdcA*. For *P. aeruginosa*, the mixture was shaken at 150 rpm for 1 min and then incubated for 2 h (26 h total) to establish the extent of mature biofilm formation. Biofilm dispersal was determined after 19 h of IPTG induction (43 h total). Comparison of these two values gave the percentage of biofilm dispersal. Similarly, for *P. fluorescens *and *R. meliloti*, biofilm formation was determined and compared after 2 h and 21.5 h of IPTG induction. At least two independent cultures were used for each strain. To remove growth effects, biofilm formation was normalized by dividing total biofilm by bacterial growth in the bulk liquid.

For BdcA dispersal in dual-species biofilms of *E. coli/R. meliloti *and *E. coli/P. aeruginosa *formed in 96-well plates, pMMB206-BdcA was mobilized using *E. coli *DH5α*/*pMMB206-BdcA/pRK2013 by adding 300 μL of turbidity 0.05 of these washed cells to the *R. meliloti *and *P. aeruginosa *biofilms formed for 24 h. After 4 h, 0.1 mM IPTG was added to produce BdcA, and cells were grown for an additional 26 h. To approximate the conjugation efficiency, biofilm cells were taken from the air/liquid biofilm (top) of each well in the 96-well plates at the end of the experiment and plated on LB, LB + rifampicin, and LB + rifampicin + chloramphenicol selective plates. For rifampicin, 20 μg/mL inhibits *E. coli, R. meliloti *is resistant to 20 μg/mL, and *P. aeruginosa *is resistant to 100 μg/mL; chloramphenicol was used to select for cells harboring pMMB206 or pMMB206-BdcA. The conjugation efficiency of *R. meliloti *and *P. aeruginosa *was calculated by comparing the number of recipient cells harboring pMMB206 or pMMB206-BdcA with the total number of recipient cells for each species. In addition, five random colonies were selected from LB plates with 100 μg/mL rifampicin and 250 μg/mL chloramphenicol (selective plates for *P. aeruginosa*/pMMB206-BdcA) and another five colonies were selected from LB plates with 20 μg/mL rifampicin and 50 μg/mL chloramphenicol (selective plates for *R. meliloti*/pMMB206-BdcA) to verify the presence of the plasmid and to identify the host. The presence of the plasmid in recipient cells was confirmed via PCR of the *bdcA *gene with the bdcAc f-primer and the bdcAc r-primer, and the bacterial species of the recipient cells was confirmed via sequencing of the 16S rRNA genes (primers are the 1492R-primer 5'-GGTTACCTTGTTACGACTT-3' and the 27F-primer 5'-AGAGTTTGATCCTGGCTCAG-3' [25]).

Similarly, for BdcA dispersal in triple-species biofilms of *E. coli*, *R. meliloti*, and *P. aeruginosa*, pMMB206-BdcA was mobilized using *E. coli *DH5α*/*pMMB206-BdcA/pRK2013 by adding 300 μL of turbidity 0.05 of these cells to existing double-species biofilms formed by *R. meliloti *and *P. aeruginosa *for 24 h. After another 11 h, 0.1 mM IPTG was added to produce BdcA, and cells were grown for an additional 24 h. To determine the percentage of each of the three strains in the biofilms, cells were taken from the air/liquid biofilm and from the liquid/bottom biofilm and diluted in LB for enumeration. The biofilm composition was determined based on the difference in rifampicin resistance of these three bacteria: 20 μg/mL inhibits *E. coli*, 100 μg/mL inhibits *R. meliloti*, and *P. aeruginosa *is resistant to 100 μg/mL.

### Flow cell biofilms and image analysis

The flow cell experiments were performed as previously described [26]. pP25-gfp [27] was used to produce the green fluorescent protein (GFP) for imaging each strain. The flow cells were inoculated with cultures at an initial turbidity at 600 nm of 0.05 at a flow rate of 10 mL/min for 2 h, then fresh LB medium with chloramphenicol and carbenicillin was added at 10 mL/min. IPTG (0.1 mM) was added to each flow cell system after 24 h, and images were taken 48 h and 96 h after IPTG addition for *P. aeruginosa *and 43 h and 91 h after IPTG addition for *R. meliloti*. Biofilm images from nine random positions were visualized with IMARIS confocal software (Bitplane, Zurich, Switzerland) and analyzed by COMSTAT confocal software as previously described [26]. Two independent cultures were used for each strain; i.e., eight flow cell experiments were conducted.

### Swarming motility, swimming motility, and EPS assays

The swarming motility of *P. aeruginosa *and *P. fluorescens *was assayed with BM-2 plates [22], while the swarming motility for *R. meliloti *was measured with 0.6% Eiken agar (Eiken Chemical Co., Tokyo, Japan) plates in LB medium with 0.5% glucose. Cells grown to a turbidity at 600 nm of ~1.0 were used for inoculation, and 0.1 mM IPTG was applied for each strain to induce the expression of *bdcA*. The swarming halo was measured after 15 h incubation for *P. aeruginosa *and *R. meliloti *and after 20 h incubation for *P. fluorescens*. Two independent cultures were used for each strain and at least three plates were used for each independent culture.

Swimming motility was performed as previously described [28]. Overnight cultures were used to inoculate the plates (1% tryptone, 0.25% NaCl and 0.3% agar), and the swimming halo was measured after 11 h for *P. aeruginosa*, after 20 h for *P. fluorescens*, and after 15 h for *R. meliloti*. Two independent cultures were used for each strain, and at least three plates were used for each independent culture.

The amount of total EPS was determined as described previously [29]. Briefly, 1 mL cell cultures grown in M9-0.4% mannitol medium were collected after 48 h and boiled in water for 10 min. The supernatants were then used for an anthrone-H_2_SO_4 _assay to determine EPS concentrations. This assay was performed with two independent cultures.

## Results

### BdcA plasmid does not affect growth

In the absence of production of BdcA, all three pairs of bacteria grew at nearly the same rate (0.5860 ± 0.0003/h for *P. aeruginosa/*pMMB206 vs. 0.571 ± 0.009/h for *P. aeruginosa/*pMMB206-BdcA at 37°C; 0.512 ± 0.005/h for *P. fluorescens/*pMMB206 vs. 0.506 ± 0.002/h for *P. fluorescens/*pMMB206-BdcA at 30°C; and 0.511 ± 0.0008/h for *R. meliloti/*pMMB206 vs. 0.51 ± 0.01/h for *R. meliloti/*pMMB206-BdcA at 30°C) so the presence of *bdcA *on the plasmid does not affect growth. Hence, the bacteria are expected to form similar amounts of biofilm prior to production of BdcA for biofilm dispersal.

### BdcA increases swarming and swimming motility and decreases EPS production

**Figure 2:**
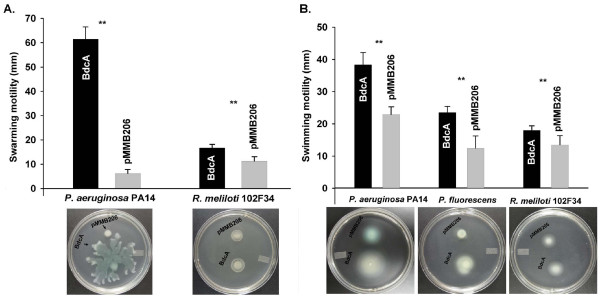
**BdcA increases swarming and swimming motility. (A) **Swarming motility of *P. aeruginosa *PA14 and *R. meliloti *102F34 with BdcA production. The *P. aeruginosa *BM-2 plates were incubated at 37°C with 250 μg/mL chloramphenicol for 15 h, and the *R. meliloti *Eiken agar plates were incubated at 30°C with 50 μg/mL chloramphenicol for 15 h. **(B) **Swimming motility of *P. aeruginosa, P. fluorescens*, and *R. meliloti *102F34. The *P. aeruginosa *plates were incubated at 37°C with 250 μg/mL chloramphenicol for 11 h, the *P. fluorescens *plates were incubated at 30°C with 250 μg/mL chloramphenicol for 20 h, and the *R. meliloti *plates were incubated at 30°C with 50 μg/mL chloramphenicol for 15 h. Black bars show the motility for strains expressing *bdcA *via pMMB206-BdcA and the gray bars indicate the control strains with pMMB206. For each strain, 0.1 mM IPTG was added to induce *bdcA *expression. ** indicates statistical significant difference as determined by a Student's t-test (*p *< 0.05). Error bars indicate the standard deviation of two independent cultures.

To investigate the impact of BdcA production on cell physiology, we assayed cell motility and EPS production since these phenotypes are controlled by c-di-GMP [13]. Production of BdcA increased both the swarming of *P. aeruginosa *and *R. meliloti *(*P. fluorescens *did not swarm under these conditions) (Figure 2A) as well as increased the swimming of *P. aeruginosa, P. fluorescens*, and *R. meliloti *(Figure 2B). Furthermore, EPS production was reduced in all three strains (1.3 ± 0.3-fold for *P. aeruginosa*, 1.8 ± 0.2-fold for *P. fluorescens*, and 1.4 ± 0.1-fold for *R. meliloti*) upon BdcA production. These phenotypes are consistent with reduced c-di-GMP concentrations in these cells in the presence of BdcA [13,22].

### BdcA increases biofilm dispersal in microtitre plates

**Figure 3:**
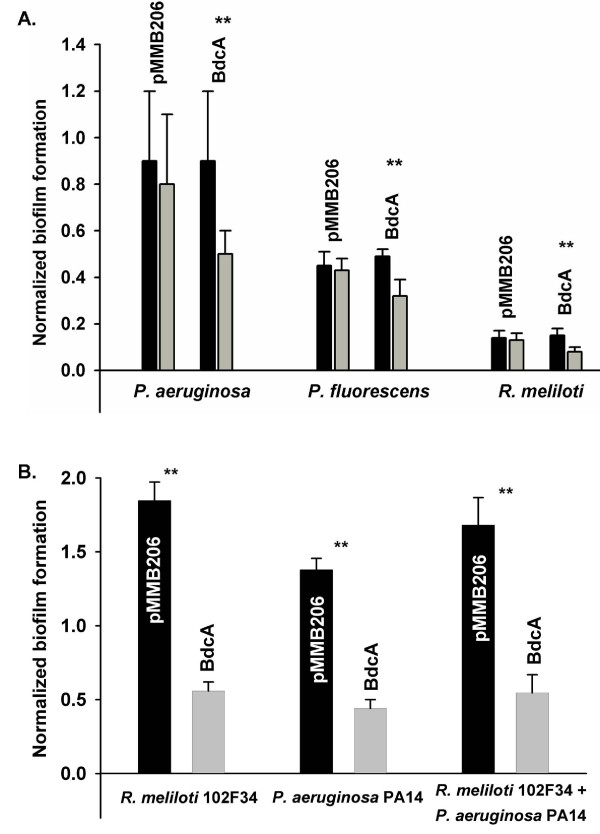
**BdcA disperses biofilms in static biofilm tests with 96-well plates. (A) **Strains as in the Figure 2 caption. Black bars indicate mature biofilm formation (2 h after IPTG induction for each strain), and the gray bars show biofilm formation after dispersal (19 h after IPTG induction for *P. aeruginosa/*pMMB206-BdcA and 21.5 h after IPTG induction for *P. fluorescens/*pMMB206-BdcA and *R. meliloti/*pMMB206-BdcA). For all three strains, 0.1 mM IPTG was added after 24 h incubation to induce *bdcA *expression. The *P. aeruginosa *biofilm was formed at 37°C in LB with 250 μg/mL chloramphenicol, the *P. fluorescens *biofilm was formed at 30°C in LB with 250 μg/mL chloramphenicol, and the *R. meliloti *biofilm was formed at 30°C in LB with 50 μg/mL chloramphenicol. pMMB206 indicates cells with this plasmid, and BdcA indicates cells containing pMMB206-BdcA. **(B) **For the dual-species biofilms, *R. meliloti *(30°C) and *P. aeruginosa *(37°C) biofilms were formed in LB for 24 h then *E. coli *DH5α*/*pMMB206-BdcA/pRK2013 was added. The mixed biofilm was developed for 4 h, then IPTG was added for 26 h to produce BdcA. For the triple species biofilm, the *R. meliloti *and *P. aeruginosa *biofilm was formed in LB for 24 h at 30°C, then *E. coli *DH5α*/*pMMB206-BdcA/pRK2013 was added. The mixed biofilm was developed for 11 h, then IPTG was added for another 24 h to produce BdcA. Normalized biofilm is indicated by dividing biofilm formation by cell turbidity. pMMB206 (black bars) indicates cells with this plasmid, and BdcA (gray bars) indicates cells containing pMMB206-BdcA. ** indicates statistical significant difference as determined by a Student's t-test (*p *< 0.05). Error bars indicate the standard deviation of two independent cultures.

Static biofilm dispersal tests showed that *bdcA *produced in trans caused 40% biofilm dispersal while the control strain with an empty vector had 5 to 10% biofilm dispersal under the same conditions in *P. aeruginosa*, *P. fluorescens*, and *R. meliloti *(Figure 3A). Critically, similar biofilm formation occurred prior to dispersal for all three strains; hence, production of BdcA only changed the dispersal stage of the biofilm (Figure 3A). Therefore, BdcA is active *in vivo *in strains other than *E. coli*, and BdcA can remove existing biofilms.

### BdcA increases biofilm dispersal in flow cells

To corroborate the static 96 well results, we tested biofilm dispersal with the more rigorous flow cell assay by continuously adding fresh medium to the biofilms with *P. aeruginosa *and *R. meliloti*. Critically, for both bacteria, biofilms were formed to the same extent prior to BdcA causing dispersal (Table 2). For example, for *P. aeruginosa*, after 72 h, there was slightly more biofilm biomass for the strain producing BdcA vs. the empty plasmid control and similar biofilm thickness and surface coverage (Table 2). After 67 h, nearly identical biofilm parameters were obtained with *R. meliloti*.

**Table 2:** Flow cell statistical analysis of biofilm formation via COMSTAT

Time	Biomass (μm^3^/μm^2^)	Surface coverage (%)	Average thickness (μm)	Roughness	Biomass (μm^3^/μm^2^)	Surface coverage (%)	Average thickness (μm)	Roughness
	***P. aeruginosa*/pMMB206/pP25-gfp**				***P. aeruginosa*/pMMB206-BdcA/pP25-gfp**			
72 h	3 ± 1	36 ± 7	6 ± 3	0.5 ± 0.3	4 ± 1	37 ± 9	8 ± 3	0.5 ± 0.2
120 h	3.1 ± 0.9	37 ± 8	7 ± 3	0.5 ± 0.2	0.2 ± 0.1	13 ± 4	0.5 ± 0.4	1.6 ± 0.2
	***R. meliloti */pMMB206/pP25-gfp**				***R. meliloti */pMMB206-BdcA/pP25-gfp**			
67 h	3 ± 1	34 ± 8	6 ± 2	0.6 ± 0.3	3 ± 1	40 ± 10	6 ± 2	0.7 ± 0.2
115 h	2.8 ± 0.7	30 ± 6	6 ± 2	0.5 ± 0.1	0.4 ± 0.3	17 ± 5	0.9 ± 0.7	1.3 ± 0.3

For *P. aeruginosa*, cultures were grown in LB with 250 μg/mL chloramphenicol and 50 μg/mL carbenicillin for 24 h, then *bdcA *expression was induced by adding 0.1 mM IPTG. Data were collected at 72 h (48 h after IPTG addition) and 120 h (96 h after IPTG addition). For *R. meliloti*, cultures were grown in LB with 50 μg/mL chloramphenicol and 50 μg/mL carbenicillin for 24 h, then *bdcA *expression was induced by adding 0.1 mM IPTG. Data were collected at 67 h (43 h after IPTG addition) and 115 h (91 h after IPTG addition). Data are the average of two independent cultures for each strain.

**Figure 4:**
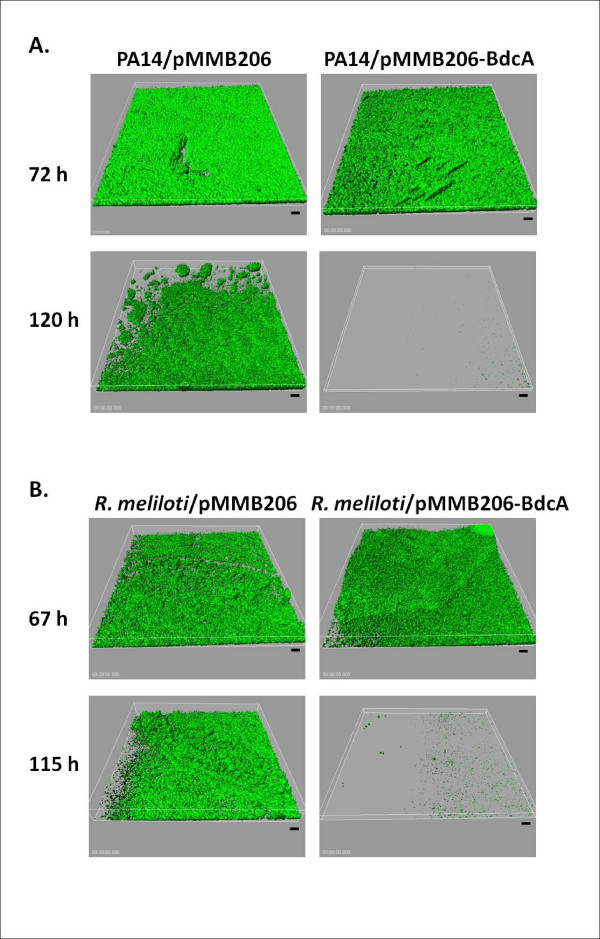
**BdcA disperses *P. aeruginosa *and *R. meliloti *biofilms in flow cell experiments**. Representative IMARIS images of *P. aeruginosa*/pMMB206-BdcA/pP25-gfp and *P. aeruginosa*/pMMB206/pP25-gfp flow cell biofilm formation after 72 h and 120 h of incubation with LB medium at 37°C **(A)**. IMARIS images of *R. meliloti*/pMMB206-BdcA/pP25-gfp and *R. meliloti*/pMMB206/pP25-gfp flow cell biofilm formation after 67 h and 115 h of incubation with LB medium at 30°C **(B)**. After forming biofilms in LB for 24 h, IPTG (0.1 mM) was added to induce BdcA production from pMMB206-BdcA. Each strain has pP25-gfp for producing GFP to visualize the biofilms, and carbenicillin (50 μg/mL) was added to retain pP25-gfp. Chloramphenicol (250 μg/mL for *P. aeruginosa *and 50 μg/mL for *R. meliloti*) was used to retain the pMMB206-based plasmids. Scale bars represent 10 μm.

Production of BdcA caused a 7-fold increase in biofilm dispersal (based on biomass) for *R. meliloti *and 15-fold increased biofilm dispersal in *P. aeruginosa *(Table 2 and Figure 4). For both strains, the biofilms were almost completely removed for the cells producing BdcA while the empty plasmid control maintained a robust biofilm (Figure 4). Hence, BdcA is extremely effective in causing biofilm dispersal in Gram-negative bacteria other than *E. coli*.

### BdcA causes dispersal in mixed-species biofilms

To demonstrate that BdcA may be added to a mixed-species biofilm via conjugation, 96-well static biofilm cultures were used to form biofilms of *R. meliloti *and *P. aeruginosa*. After 24 h, BdcA was added to these biofilms by conjugating pMMB206-BdcA from *E. coli *DH5α*/*pMMB206-BdcA/pRK2013; *E. coli *DH5α*/*pMMB206/pRK2013 was used as the negative control which conjugates but does not provide BdcA. Upon producing BdcA for 26 h, total biofilm formation was reduced by 3.3 ± 0.6 for *R. meliloti *and 3.1 ± 0.6 for *P. aeruginosa *(Figure 3B). For these experiments, the efficiency of transferring pMMB206-BdcA to *R. meliloti *and *P. aeruginosa *was over 76% and over 80% respectively, probably due to vast excess of donor *E. coli *cells relative to the recipient cells of the very small biofilm when donor *E. coli *was added. After conjugation, we checked for the presence of the correct plasmid in recipient cells via PCR for the *bdcA *gene as well as identified each bacterial species via sequencing for 16S rRNA genes to confirm that the selective plating method was accurate. For both tests, all (10) cells from the selective antibiotic plates were found to have the *bdcA *plasmid (766 bp), and these cells were all determined to be either *P. aeruginosa *or *R. meliloti*, respectively.

In addition, we used the same strategy to treat an existing biofilm formed by both *R. meliloti *and *P. aeruginosa *and obtained a 3.1 ± 0.8-fold biofilm reduction based on BdcA production after conjugation from *E. coli *strain (Figure 3B). Hence, BdcA was able to disperse a multi-species biofilm that consisted of approximately 40% *P. aeruginosa *PA14, 23% *R. meliloti*, and 37% *E. coli *(the composition of control biofilm without BdcA was similar at 37% *P. aeruginosa *PA14, 26% *R. meliloti*, and 37% *E. coli*). Therefore, BdcA may be introduced into mixed biofilms via conjugation and used to reduce biofilm formation.

## Discussion

Given the near universal nature of c-di-GMP as a biofilm formation and dispersal signal, we investigated whether BdcA from *E. coli *would be effective in removing biofilms from various bacteria. To accomplish this goal, the broad host plasmid pMMB206 was used to express *bdcA *in different organisms including *P. aeruginosa, P. fluorescens*, and *R. meliloti*. As we had hoped, BdcA production increased swarming and swimming motility and reduced EPS production; these phenotypes indicate a reduced c-di-GMP concentration in these strains. The reduced c-di-GMP concentrations via BdcA proved extremely effective in dispersing biofilms including that of the opportunistic pathogen *P. aeruginosa*. This is the first report of reducing c-di-GMP via heterologous protein production to cause biofilm dispersal in these three strains. This is an important result in that it is a significant challenge to remove biofilms [30,31] since cells in biofilms are cemented in place by the secreted polymer matrix consisting of polysaccharide, protein, DNA, and lipids [2]; this matrix of the biofilm colony makes most biofilms difficult or impossible to eradicate [4].

BdcA directly reduces bacterial c-di-GMP concentrations [13], and c-di-GMP is a signal that controls physiology of diverse bacteria [32]. In *R. meliloti*, c-di-GMP controls growth, motility, EPS production, and even the interactions between bacteria and host plants [33]. In *P. aeruginosa*, c-di-GMP controls various cell activities including biofilm formation, EPS production, and aggregation [22]. Hence, BdcA may control biofilm dispersal in as many organisms as c-di-GMP functions as a second messenger. In addition, *bdcA *shows high sequence conservation with other species (including *Pseudomonas *and *Rhizobium*) and is well conserved in many bacteria [13]. Hence, BdcA may be used for dispersal in many strains. As a preliminary proof of this principle, our results demonstrate the feasibility of introducing BdcA into existing non-*E. coli *biofilms (formed by *P. aeruginosa *and *R. meliloti *individually, as well as by mixed biofilms of these two bacteria) via conjugation of a broad-host-range plasmid) to reduce total biofilm formation. Therefore, in this study, we successfully disperse biofilms of disparate strains simply by producing BdcA. Hence, through this genetic approach, a single protein may be used to remove biofilms rather than by using traditional chemical or physical means.

## List of abbreviations

c-di-GMP: cyclic diguanylate; IPTG: isopropyl-β-*D*-thiogalactopyranoside; LB: Luria-Bertani; GFP: green fluorescent protein.

## Competing interests

The authors declare that they have no competing interests.

## Authors' contributions

TKW and QM designed the study and wrote the paper. QM and GZ performed the experiments and analyzed the results.
